# Multiplexing and Demultiplexing of Aperture-Modulated OAM Beams

**DOI:** 10.3390/s25134229

**Published:** 2025-07-07

**Authors:** Wanjun Wang, Liguo Wang, Lei Gong, Zhiqiang Yang, Ligong Yang, Yao Li, Zhensen Wu

**Affiliations:** 1 School of Optoelectronic Engineering, Xi’an Technological University, Xi’an 710021, China; wangliguo@xatu.edu.cn (L.W.); gonglei@xatu.edu.cn (L.G.); yangzhiqiang@xatu.edu.cn (Z.Y.); yanglihong@xatu.edu.cn (L.Y.); liyao_xatu@163.com (Y.L.); 2 School of Physics, Xidian University, Xi’an 710021, China; wuzhs@mail.xidian.edu.cn

**Keywords:** multi-parameter multiplexing and demultiplexing, aperture carrier, OAM beams, free space with turbulence

## Abstract

**Highlights:**

**What are the main findings?**

- The aperture size carried by the orbital angular momentum could be modulated by the external variable aperture as a new information carrier.
- The field of the beams propagating through turbulence was derived and discretized with Gauss–Legendre quadrature formulas. Based on this, the demultiplexing method was improved, and the beam OAM states, amplitude, Gaussian spot radius and aperture radius were decoded.

**What are the implications of the main findings?**

- The aperture size as a new information carrier can be modulated more easily through the external variable aperture.
- Through discretization with Gauss–Legendre quadrature formulas, the information carried by the beams with an integral expression at the receiver plane can be demultiplexed.

**Abstract:**

A multiplexing method for orbital angular momentum (OAM) beams was proposed. The aperture size as a new information carrier was provided, and it could be modulated by the external variable aperture. The field of the beams propagating through turbulence was derived and discretized with Gauss–Legendre quadrature formulas. Based on this, the demultiplexing method was improved, and the beam OAM states, amplitude, Gaussian spot radius and aperture radius were decoded. Moreover, the influence of turbulence on the multiplexing parameters was also analyzed, and the decoding precision of the aperture radius was higher than that of other parameters. The aperture radius was recommended as an extra carrier for multiplexing communication. This study provides a simple method to modulate the information carried by OAM beams, and it has promising applications in large capacity laser communication.

## 1. Introduction

Vortex laser beams, widely studied for their orbital angular momentum (OAM) can be applied as an additional communication dimension to increase the information capacity [1,2,3]. To investigate the properties of OAM beams, Gaussian vortex beams (Gv) have been proposed for their simple source expression, and ability to be generated by Gaussian beams through spiral phase plates [4,5,6].

**Figure 1:**
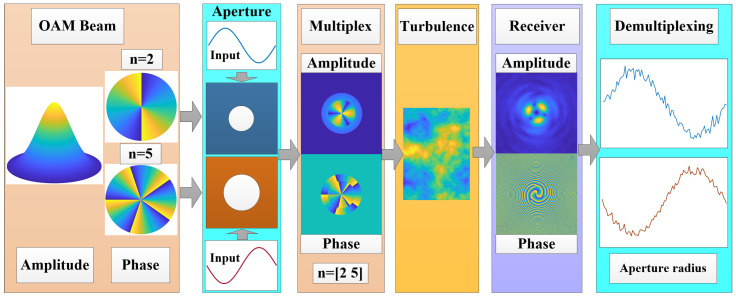
Multiplexing and demultiplexing of OAM beams in turbulence.

OAM multiplexing methods have been widely investigated to increase channel capacity [7,8,9]. Multiple coaxially vortex beams with different OAM states are a common multiplexing method, and the OAM states can be demultiplexed by Fourier decomposition of the wave-front [10,11]. Hence, the mixed methods have been studied, such as different OAM and wavelengths multiplexing [12,13]. The wavelength can be separated based on the beam divergence difference. However, this multiplexing method is limited by the finite number of available laser wavelengths, and its application in long-distance communication has not been extensively investigated. Space-division multiplexing is another way to increase communication capacity, and the information is carried by multi-off-axis beams with different OAM [14,15,16,17,18,19]. In addition, ring-shaped beams carrying different OAM states in the radial direction were investigated [20,21]. However, with these methods, it is difficult to distinguish the OAM states in long-haul communication, because there is serious overlapping and crosstalk of the beam OAM caused by beam divergence after long distance propagation [22,23,24]. To make full use of the beam spatial information, the beam width was investigated as an extra information dimension to carry the signal [25]. However, the carrier information is not readily suited to be fast modulation, as it is limited by the modulation response time of the source intensity distribution. Under this circumstance, this study proposes a multiplexing method using truncated Gv beams, with carrier parameters that are easier to modulate with external variable apertures. As Figure 1 shows, coaxial sub Gv beams with different OAM states are propagated through different size apertures, respectively, and the aperture radius is selected as the input. Subsequently, the multiplexing beam propagates through the turbulence, and demultiplexed at the receiver plane.

In this study, a multiplexing method for Gv beams is proposed, and the OAM states, amplitude, Gaussian spot radius, and aperture radius as new information carriers are decoded. This study is conducive to the communication link design and application of large-capacity OAM multiplexing communication.

## 2. Materials and Methods

### 2.1. Gaussian Vortex Beam

Gaussian vortex beams (Gv) as one type of OAM beams have been widely studied, and their field distribution with radial coordinates *r* and $\varphi_{r}$ is expressed as Equation (1) [1,6].

(1) $$U_{0}(r,\varphi_{r})=A\exp(-k\alpha r^{2})\exp(-in\varphi_{r}),$$

where *n* is the order of the Gv beams, *k* is the wave number, λ is the wavelength, and k=2π/λ. Here, $\alpha =1/kw_{0}^{2}+i/(2F_{0})$, where $w_{0}$ is the Gaussian spot radius and $F_{0}$ is the focusing parameter. *A* is the beam amplitude. In this study, the beams are collimated beams and $F_{0}=\infty$.

The aperture is frequently applied to constrain the beam energy distribution. The truncated Gv beam was provided as Equation (2).

(2) $$U_{0}(r,\varphi_{r})=A\exp(-k\alpha r^{2})\exp(-in\varphi_{r})\omega (r),$$

where ω(r) is the window function. This function is expressed as Equation (3).

(3) $$\omega (r)=\left\{\begin{array}{cc} 1, & \;r\leq R_{a}\\ 0, & \;r>R_{a} \end{array}\right.,$$

where *R_a_* is the size of the aperture radius.

Based on the extended Huygens–Fresnel principle, as given in Equation (21) in Section 7.3 of Ref. [26], the beam field in the free space is expressed as Equation (4).

(4) $$U_{FS}(r,L)=-\frac{ikA}{2\pi L}\exp(ikL)\int \int_{-\infty }^{\infty }d^{2}sU_{0}(s,0)\exp\left(\frac{ik{\left|s-r\right|}^{2}}{2L}\right),$$

where *L* is the propagation distance, and $U_{0}(s,0)$ is the field of the Gv beams with column coordinates vector s and L=0.

Substituting Equation (1) into Equation (4), and applying Equation (2) from Section 3.937 of Ref. [27], the field can be simplified as shown in Equation (5).

(5) $$U_{FS}(r,L)=-\frac{ikA}{L}\exp\left(ikL+\frac{ikr^{2}}{2L}\right)\exp\left(-in\varphi_{r}\right)\int_{0}^{\infty }dss\exp\left(-k\alpha s^{2}+\frac{iks^{2}}{2L}\right)i^{n}J_{n}\left(-\frac{k}{L}sr\right),$$

A formula to simplify Equation (5) is rewritten as Equation (6) based on Equation (7) in Section 6.631 of Ref. [27].

(6) $$\int_{0}^{\infty }x\exp(-ax^{2})J_{n}(bx)dx=\frac{\sqrt{\pi }b}{8a^{3/2}}\exp\left(-\frac{b^{2}}{8a}\right)\left[I_{\frac{1}{2}v-\frac{1}{2}}\left(-\frac{b^{2}}{8a}\right)-I_{\frac{1}{2}v+\frac{1}{2}}\left(-\frac{b^{2}}{8a}\right)\right],$$

Substituting Equation (6) into Equation (5), the field of the Gv beam in free space can be derived as Equation (7). And Equation (7) expresses the beam field without the influence of turbulence.

(7) $$\begin{array}{ll} U_{FS}(r,L) & =-\frac{A\sqrt{2\pi ikL}i^{n}r}{4L{\left(1+2i\alpha L\right)}^{3/2}}\exp\left[ikL+\frac{ikr^{2}\left(1+4i\alpha L\right)}{4L\left(1+2i\alpha L\right)}\right]\exp\left(-in\varphi_{r}\right)\\ & \times \left\{I_{\frac{1}{2}n-\frac{1}{2}}\left[\frac{ikr^{2}}{4L\left(1+2i\alpha L\right)}\right]-I_{\frac{1}{2}n+\frac{1}{2}}\left[\frac{ikr^{2}}{4L\left(1+2i\alpha L\right)}\right]\right\} \end{array},$$

The field distribution of the beams propagating through the turbulence can be expressed as Equation (8) based on the Rytov approximation under weak fluctuation conditions [25,26].

(8) $$U(r,L)=U_{FS}(r,L)\exp\left[\Psi (r,L)\right],$$

Based on the extended Huygens–Fresnel principle, the mean of the field is derived as Equation (9) [26].

(9) $$\langle U(r,L)\rangle =U_{FS}(r,L)\langle \exp\left[\Psi (r,L)\right]\rangle =U_{FS}(r,L)\exp\left[E_{1}(r,r)\right],$$

where *E*_1_ is the turbulence moment, and expresses as Equation (10), $\Phi_{n}(\kappa )$ is the power spectrum, and the von Karman spectrum is used as Equation (11) [26].

(10) $$E_{1}(r,r)=-\pi k^{2}\int_{0}^{L}d\eta \int \int_{-\infty }^{\infty }d^{2}\kappa \Phi_{n}(\kappa ),$$

(11) $$\Phi_{n}(\kappa )=0.033C_{n}^{2}\frac{\exp\left(-\kappa^{2}/\kappa_{m}^{2}\right)}{{\left(\kappa^{2}+\kappa_{0}^{2}\right)}^{11/6}},$$

where *l*_0_ and *L*_0_ is the inner and outer scales of the turbulence, and $\kappa_{m}=5.92/l_{0}$, $\kappa_{0}=2\pi /L_{0}$, respectively. In addition, $C_{n}^{2}$ is the refractive index structure constant.

Substituting Equation (7) into Equation (8), the field of Gv beams in turbulence can be expressed as Equation (12).

(12) $$U(r,L)=a_{n}(r)\exp(-in\varphi_{r}),$$

where $a_{n}(r)$ is the complex amplitude of the beams and expresses as Equation (13).

(13) $$\begin{array}{l} a_{n}(r)=-A\frac{\sqrt{2\pi ikL}i^{n}r}{4L{\left(1+2i\alpha L\right)}^{3/2}}\exp\left[ikL+\frac{ikr^{2}\left(1+4i\alpha L\right)}{4L\left(1+2i\alpha L\right)}\right]\\ \left\{I_{\frac{1}{2}n-\frac{1}{2}}\left[\frac{ikr^{2}}{4L\left(1+2i\alpha L\right)}\right]-I_{\frac{1}{2}n+\frac{1}{2}}\left[\frac{ikr^{2}}{4L\left(1+2i\alpha L\right)}\right]\right\}\exp\left[\Psi (r,L)\right] \end{array},$$

Using the same method, the field of truncated Gv beams could be expressed as noted in Equation (14) based on Equation (5).

(14) $$\begin{array}{ll} U(r,L) & =-A\exp\left[\Psi (r,L)\right]\frac{i^{n+1}k}{L}\exp\left(ikL+\frac{ikr^{2}}{2L}\right)\exp\left(-in\varphi_{r}\right)\\ & \int_{0}^{R_{a}}dss\exp\left(-k\alpha s^{2}+\frac{iks^{2}}{2L}\right)J_{n}\left(-\frac{k}{L}sr\right) \end{array},$$

The parameters in the integral were hard to solve, and integral was discretized to polynomial terms based on the Gauss-Legendre quadrature [28]. The beam field is expressed as Equation (15).

(15) $$\begin{array}{ll} U(r,L) & =-A\exp\left[\Psi (r,L)\right]\frac{i^{n+1}k}{L}\exp\left(ikL+\frac{ikr^{2}}{2L}\right)\exp\left(-in\varphi_{r}\right)\\ & \sum_{l=1}^{M}A_{p_{l}}p_{l}\exp\left(-k\alpha p_{l}^{2}+\frac{ikp_{l}^{2}}{2L}\right)J_{n}\left(-\frac{k}{L}p_{l}r\right) \end{array},$$

where $p_{l}$ and $A_{p_{l}}$ are the nodes and weights of the Gauss–Legendre quadrature, respectively. The total number of nodes is *M*. In this study, the number of nodes is 17.

The original nodes and weights could only be applied to solve integrals in interval [-1, 1]. Therefore, the nodes and weights should be transformed to corresponding values in the interval [0, *R_a_*]. The transformation relationship are expressed as Equations (16) and (17).

(16) $$p_{l}=0.5R_{a}P_{l}+0.5R_{a},$$

(17) $$A_{p_{l}}=0.5R_{a}A_{P_{l}},$$

where $P_{l}$ and $A_{P_{l}}$ are the original nodes and weights, respectively, which can be obtained from Table 25.4 of Ref. [28].

Substituting Equations (16) and (17) into Equation (15), the beam field of the truncated Gv beam is expressed as Equation (18).

(18) $$\begin{array}{ll} U(r,L) & =-\frac{A\exp\left[\Psi (r,L)\right]}{4}\frac{i^{n+1}k}{L}\exp\left(ikL+\frac{ikr^{2}}{2L}\right)\exp\left(-in\varphi_{r}\right)\\ & \times \sum_{l=1}^{M}A_{P_{l}}\left(P_{l}+1\right)R_{a}^{2}\exp\left[-\frac{k\alpha }{4}{\left(P_{l}+1\right)}^{2}R_{a}^{2}+\frac{ik}{8L}{\left(P_{l}+1\right)}^{2}R_{a}^{2}\right]J_{n}\left[-\frac{k}{2L}\left(P_{l}+1\right)R_{a}r\right] \end{array},$$

### 2.2. Beam Center Location Correction

The beam field sampled using the phase screen method is expressed in Cartesian coordinates [29,30]. To decode the carrier information, the beam field must be preprocessed and transformed to polar coordinates starting from the beam center. When beams propagate through the turbulence, the beam is distorted, and the beam center wanders around the initial location [26,31]. Therefore, based on the cylindrical symmetry of the beam, a method to obtain the diffused beam center was provided.

The distorted beam center location was approximated by a circle center composed by the average radius of the beam intensity in different directions. The average radius in the direction $\varphi_{j}$ is expressed as Equation (19).

(19) $${\bar{R}}_{j}=\frac{\sum_{r_{i}}\sqrt{{\left(r_{i}\cos\varphi_{j}-x_{0}\right)}^{2}+{\left(r_{i}\sin\varphi_{j}-y_{0}\right)}^{2}}I(r_{i}\cos\varphi_{j},r_{i}\sin\varphi_{j})}{\sum I(r_{i}\cos\varphi_{j},r_{i}\sin\varphi_{j})},$$

where $I(r_{i},\varphi_{j})$ is intensity at the location in direction $\varphi_{j}$ with the beam radius $r_{i}$. The relationship between the polar coordinates and the Cartesian coordinates is expressed as Equation (20). Here, $\left(x_{0},y_{0}\right)$ is the initial beam center location, and it is initialized by the centroid expressed as Equation (21). The number of the directions $\varphi_{j}$ depends on the rotational symmetry of the beam intensity.

(20) $$\begin{array}{l} x=r_{i}\cos\varphi_{j}\\ y=r_{i}\sin\varphi_{j} \end{array},$$

(21) $$x_{0}=\frac{\sum xI(x,y)}{\sum I(x,y)},y_{0}=\frac{\sum yI(x,y)}{\sum I(x,y)},$$

where I(x,y) is the beam intensity, and which is equal to the square of the magnitude of the field.

The average radius and the beam center satisfies the equation as Equation (22).

(22) $${\left({\bar{R}}_{j}\cos\varphi_{j}-x_{k}\right)}^{2}+{\left({\bar{R}}_{j}\sin\varphi_{j}-y_{k}\right)}^{2}=R_{k}^{2},$$

where $R_{k}$ is the circle radius during the *k*th iteration.

The new center $\left(x_{k},y_{k}\right)$ could be solved through using multiple group equations in different direction $\left({\bar{R}}_{j},\varphi_{j}\right)$ as Equation (22) with the least squares method.

The steps are repeated until the error between two center positions less than the tolerance as Equation (23). At this time, $\left(x_{k},y_{k}\right)$ is the center position of the distorted beam.

(23) $${\left(x_{k}-x_{k-1}\right)}^{2}+{\left(y_{k}-y_{k-1}\right)}^{2}<\varepsilon ,$$

where ε is the threshold, and it is one pixel in general.

### 2.3. Multi-Parameter Demultiplexing Method

The complex amplitude is calculated using the inverse fast Fourier transform to the field intensity [10,11], and it is expressed as Equation (24).

(24) $$a_{n}(r)=F^{-1}\left[U_{PS}(r_{x},r_{y},L)\right],$$

where $U_{PS}(r_{x},r_{y},L)$ is field sampled using the phase screen method [24,29], and it was transformed from the Cartesian coordinates to polar coordinates.

The field intensity sampled using the phase screen method as noted in Equation (24), is equal to that derived based on the extended Huygens–Fresnel principle as Equations (12) and (18). Therefore, the multiplexing parameters could be solved based on the equations of the complex amplitude at different radii [25].

The beam amplitude and the beam width parameters in Equation (18) are selected to carry the information, and are represented as Equation (25).

(25) $$a_{n}(r)=C_{1}r\exp\left[\frac{ik\left(1+4iC_{2}L\right)}{4L\left(1+2iC_{2}L\right)}r^{2}\right]\left\{I_{\frac{1}{2}n-\frac{1}{2}}\left[\frac{ikr^{2}}{4L\left(1+2iC_{2}L\right)}\right]-I_{\frac{1}{2}n+\frac{1}{2}}\left[\frac{ikr^{2}}{4L\left(1+2iC_{2}L\right)}\right]\right\},$$

where *C*_1_, and *C*_2_ are the parameters to be decoded.

Given the $a_{n}(r)$ along the radius, complex parameters *C*_1_ and *C*_2_ can be obtained by searching for the optimal solution with the nonlinear curve-fitting method in the least-squares sense. The decoded parameters are complex numbers. Their magnitudes are used approximate to the information carried by the beam as Equations (26) and (27) [25].

(26) $$A_{N}=A\exp\left[\Psi (r,L)\right]=\left|-\frac{4L{\left(1+2iC_{2}L\right)}^{3/2}}{\sqrt{2\pi kL}}C_{1}\right|,$$

(27) $$w_{0}=\sqrt{k^{-1}C_{2}^{-1}},$$

where | | represents the magnitude of the complex number in this section. The turbulence influence Ψ(L) is unknown. Therefore, only the normalized amplitude *A_N_* can be decoded.

The Gaussian spot radius and the aperture radius are parameters related to the beam width. To investigate the decoding precision of the beam width, only one radius was varied, and another was set as a constant.

For the truncated Gv beam, the complex amplitude could be expressed as noted in Equation (28) based on Equation (18).

(28) $$a_{n}(r)=C_{3}\exp\left(\frac{ikr^{2}}{2L}\right)\sum_{l=1}^{M}A_{p_{l}}p_{l}\exp\left(-C_{4}p_{l}^{2}+\frac{ikp_{l}^{2}}{2L}\right)J_{n}\left(-\frac{k}{L}p_{l}r\right),$$

where *C*_3_, and *C*_4_ are parameters to be decoded. The input parameters are expressed as Equation (29).

(29) $$A_{N}=\frac{L}{k}\left|C_{3}\right|,\;w_{0}=\sqrt{\left|C_{4}^{-1}\right|},$$

The beam wave-front varied with the diffraction aperture size, and the aperture was applied as a carrier. The complex amplitude of the truncated Gv beam with the aperture radius to be decoded is expressed as Equation (30).

(30) $$\begin{array}{l} a_{n}(r)=\frac{C_{3}}{4}\exp\left(\frac{ikr^{2}}{2L}\right)\sum_{l=1}^{M}A_{P_{l}}\left(P_{l}+1\right)C_{6}^{2}\\ \exp\left(-\frac{k\alpha {\left(P_{l}+1\right)}^{2}}{4}C_{6}^{2}+\frac{ik{\left(P_{l}+1\right)}^{2}}{8L}C_{6}^{2}\right)J_{n}\left(-\frac{k\left(P_{l}+1\right)r}{2L}C_{6}\right) \end{array},$$

where *C*_5_, and *C*_6_ are parameters to be decoded, and input parameters are expressed as Equation (31).

(31) $$A_{N}=\frac{L}{k}\left|C_{5}\right|,\;R_{a}=\left|C_{6}\right|,$$

In general, the truncated window length $R_{a}$ can be controlled by modulating the size of the external aperture radius, but the Gaussian spot radius $w_{0}$ is difficult to modulate using the external equipment.

To investigate the decoding precision, the beam field was calculated using the phase screen method to generate sample data [24,25].

## 3. Results and Discussion

In this study, without special annotation, the number of the sampling points around one circumference was m = 180, and the wavelength was λ=1.55 μm. The turbulence inner scale was *l*_0_ = 0.02 m, and the outer scale was *L*_0_ = 2 m. The phase screen size was 1.2 m, and the number of sampling points of the phase screen was 1024. The statistical parameters such as the mean and coefficient of variation, were the average values of 1000 samples.

**Figure 2:**
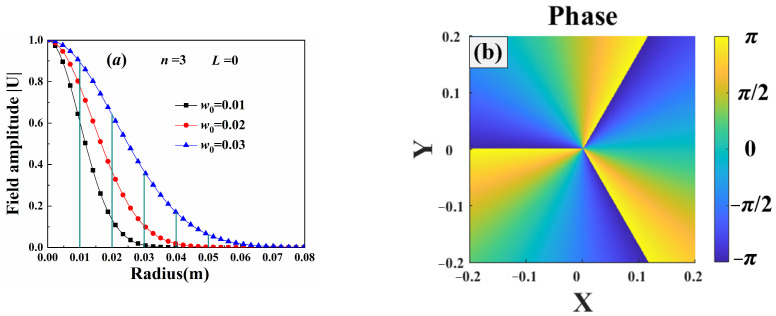
Source of third-order Gv beams: (**a**) field amplitude, (**b**) phase.

Figure 2 shows the field amplitude and phase of the third-order Gv beam at the source plane. The beam exhibited a cylindrically symmetric distribution, as shown in Equation (1), and the amplitude decreased along the beam radius. The max amplitude was noted at the beam center, and the beam amplitude was close to zero at the beam edge. The beam phase displayed a periodic distribution, with the angular period equal to the beam order.

**Figure 3:**
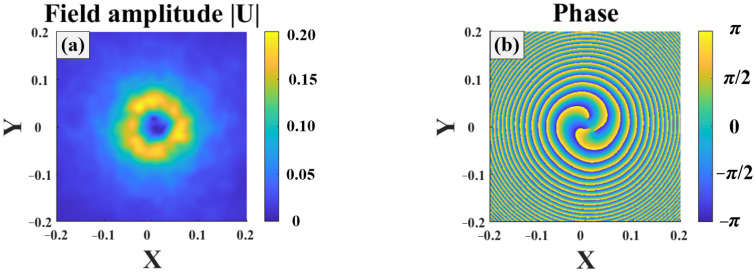
Third order Gv beam propagating in turbulence: (**a**) field amplitude, (**b**) beam phase.

Figure 3 illustrates the Gv beam propagating in turbulence. Here, the turbulence strength was $C_{n}^{2}=1\times 10^{-14}$ and $w_{0}=0.02$. The propagation distance was 1000 m. The turbulence caused beam distortion, which is one of the major factors causing the decoding error. The distribution of the Gv beam field was much different from that at the beam source. The beam spread and diffused, transforming into a hollow beam, as shown in Figure 3a. Moreover, distribution of the beam phase exhibited an uneven spiral distribution, as shown in Figure 3b, after the beam propagated a certain distance.

**Figure 4:**
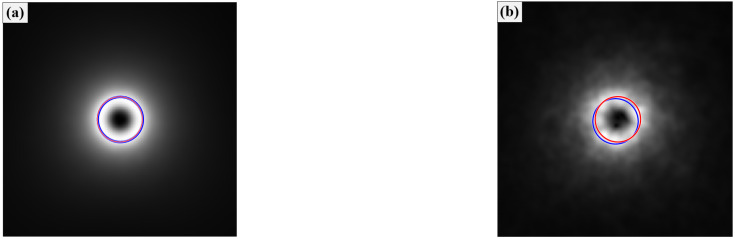
Position circles with $w_{0}=0.02$, n=3: (**a**) $C_{n}^{2}=1\times 10^{-16}$, (**b**) $C_{n}^{2}=1\times 10^{-14}$.

Figure 4 shows the position circles to represent the location of the beam center. The center of the red circle was calculated using Equation (23), and the center of the blue circle was obtained using the centroid method. When the turbulence strength was small, the locations of these two circles were almost the same, as shown in Figure 4a. Due to the uneven distribution of the beam intensity caused by the strong strength of the turbulence, as noted in Figure 3a, the center location calculated using the centroid method deviated from the theoretical center position, as shown in Figure 4b. In general, the theoretical instantaneous center position was hard to obtain due to measurement error and atmospheric effects. Thus, this method using Equation (23) could be used for the beam center calculation and applied to the center correction.

**Figure 5:**
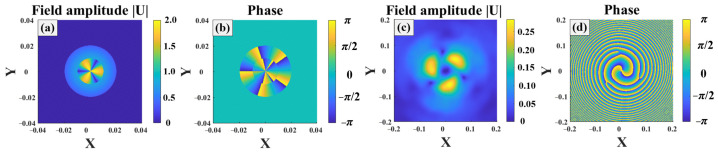
Multiplex Gv beams propagating in turbulence with *n* = [2, 5]: (**a**) field amplitude at the beam source, (**b**) phase at the beam source, (**c**) field amplitude at distance *L* = 1000, (**d**) phase at distance *L* = 1000.

Figure 5 shows multiplex Gv beams propagating in turbulence. The beam was composed of coaxial second-order and fifth-order sub-beams. The turbulence strength was $C_{n}^{2}=1\times 10^{-15}$ and $w_{0}=0.02$. The aperture radius of the second-order sub-beam was 0.01, and that of fifth-order sub-beam was 0.02. The intensity overlapping area of second-order and fifth-order sub-beams exhibited a period distribution, and the angular frequency was equal to the order difference of the sub-beams [32]. At the propagation distance *L* = 1000, the beam amplitude maintained the same angular frequency, but the boundaries between different order sub-beams were no longer distinct, as shown in Figure 5c.

**Figure 6:**
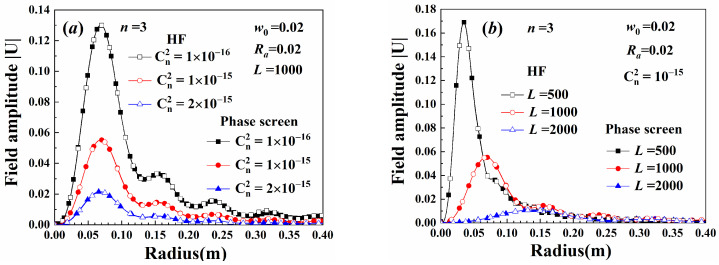
Average amplitude of the beam field: (**a**) variation with turbulence strength, (**b**) variation with propagation distance.

Figure 6 shows the average amplitude of the beam field. In the figure, HF represents the result calculated using the extended Huygens–Fresnel principle, and phase screen represents the value computed using the phase screen method. The amplitudes calculate using these two methods coincided well. The mean of the field amplitude decreased as the turbulence strength increased. The beam diverges when it propagates through the turbulence, and the max amplitude is reduced as the propagation distance increases. When the turbulence strength is large, the statistic moments cannot converge to a constant given the chaotic properties of the turbulence. A bias between the theoretical method and the phase screen method was observed.

**Figure 7:**
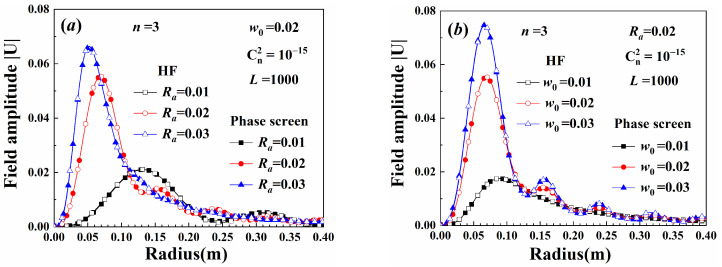
Average amplitude of truncated Gv beams: (**a**) variation with truncated aperture radius, (**b**) variation with Gaussian spot radius.

Figure 7 shows the average amplitude of the truncated Gv beam. Significant differences were observed in the field distribution of Gv beams with varying truncated radii, as shown in Figure 7a. When the width of the beam truncated window function was sufficiently large, the beam field was close to that of Gv beams without a truncated aperture. As the truncated aperture radius or the Gaussian spot radius increased, the beam divergence was reduced, and its amplitude increased. Therefore, beams with larger widths were less affected by turbulence. Given the same difference in the abscissa of the max amplitude, the variation of the truncated aperture radius was less than that caused by changes in the Gaussian spot radius.

**Figure 8:**
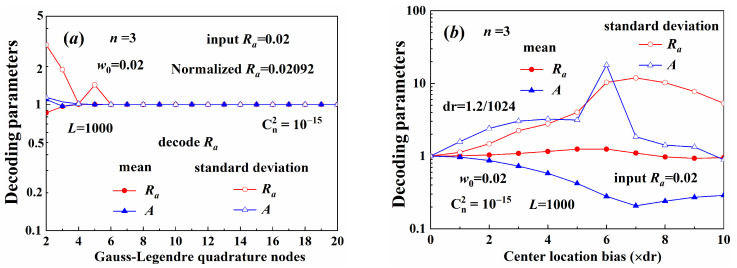
(**a**) Decoding precision varying with the number of Gauss–Legendre quadrature notes, (**b**) Decoding precision varying with a center bias.

Figure 8a shows the decoding precision calculated using Gauss–Legendre quadrature method with different node numbers. The decoding parameters were normalized using the value calculated with the Gauss–Legendre quadrature method with 20 nodes. When the node number was greater than 6, the decoding parameters were close to a constant, and the error between the decoding aperture radius and input was less than 5%. To obtain high decoding precision, the number of nodes in the Gauss–Legendre quadrature method was set to 17. Figure 8b shows the decoding precision of the beam with a bias between the center of the input beam and that of the decoding beam center. When the center bias was smaller than 1 one pixel (about 0.001), the decoding precision of the aperture radius was less than 10%, and the demultiplexing method could be applied to decode the signal. For the beams with two sub-beams, when the axis alignment precision in a multi-coaxial configuration was larger than one pixel (about 0.001), the demultiplexing method was unable to decode the beam parameters.

**Figure 9:**
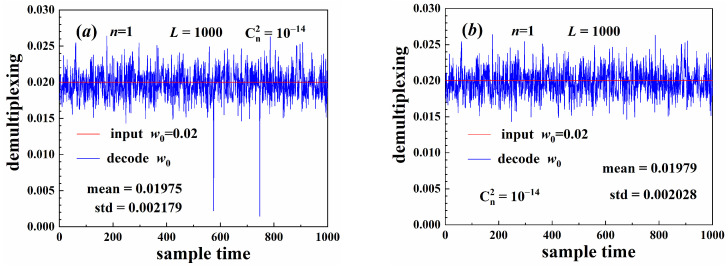
Parameter decoding: (**a**) without beam center correction, (**b**) with beam center correction.

Figure 9 illustrates the beam width parameter decoding results. The time-varying turbulence was sampled with different random seeds at different times. When beams propagated through the turbulence, the carrier signal experienced different perturbations at different sampling times. The beam center wandered when the beams propagated through the turbulence [26,31], causing decoding error with a large bias between the decoding result and the input information, as shown in Figure 9a. Using beam center correction, this bias could be reduced to appropriate levels, as shown in Figure 9b. The turbulence caused beam distortion and diffusion. The circumferential distribution of the beam intensity was also uneven, as shown in Figure 3a. This is the major factor that caused the decoding error. Although the center of the distorted beam was close to the theoretical value, decoding error may also occur. Therefore, the beam center correction method was more appropriate for the error correction.

**Figure 10:**
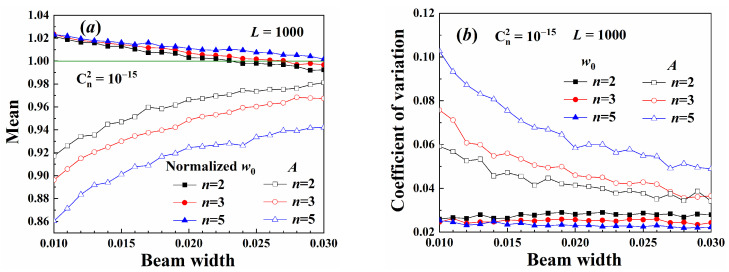
Parameter decoding: (**a**) mean, (**b**) coefficient of variation.

Figure 10 shows the decoding results of the demultiplexing method. Low-order and wider beams diverged slowly, and their maximum intensity was higher. Therefore, the average amplitude of the low-order beam was larger and closer to the input amplitude, as shown in Figure 10a. The coefficient of variation of the low-order beam amplitude was also small, as shown in Figure 10b. The demultiplexing method could only decode the normalized amplitude, which contained the perturbation caused by the turbulence, as noted in Equation (26). Therefore, the Gaussian spot radius was more resistant to turbulence-induced distortions than the normalized amplitude. The decoding precision of the beam width was higher than that of the amplitude.

**Figure 11:**
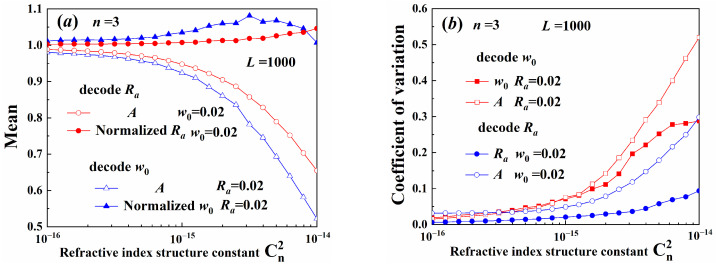
Demultiplexing width parameters: (**a**) mean, (**b**) coefficient of variation.

Figure 11 illustrates the decoding results of the different width beams. The decoding precision of the aperture radius of the constant Gaussian spot radius beams was higher than that of the Gaussian radius of the constant aperture radius beams. Moreover, the coefficient of variation was also smaller. As illustrated in Figure 7, the radial variation of the maximum amplitude in beams with variable aperture was larger than in beams with variable Gaussian spot size. Therefore, the decoding precision was more sensitive to the truncated aperture radius. The aperture radius, as described in Equation (30), is recommended as extra information carrier and can be demultiplexed with high precision.

**Figure 12:**
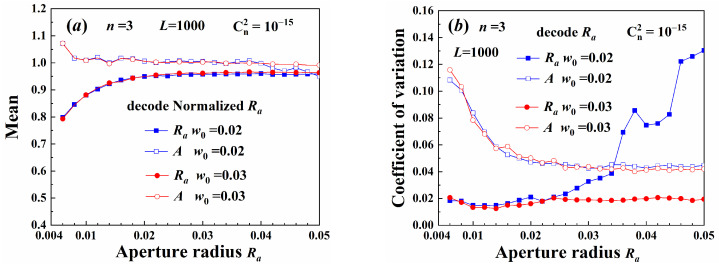
Demultiplexing parameters: (**a**) mean, (**b**) coefficient of variation.

Figure 12 shows the decoding parameters of different width beams. There was a parameter decoding error for beams with a small truncated aperture radius. This occurred because the figure resolution simulated by the phase screen method was 0.0012, which was relatively large, making it difficult to accurately construct beams with small aperture radii at the source plane. Conversely, when the aperture radius was much larger than the Gaussian spot radius, a decoding error also occurred. Since the field distribution of beams with large apertures closely resembled that of beams without any aperture, as shown in Figure 2a, the aperture radius became difficult to distinguish. Therefore, the aperture radius used as a carrier should be smaller than the beam spot size and positioned within the bright area of the beam source.

**Figure 13:**
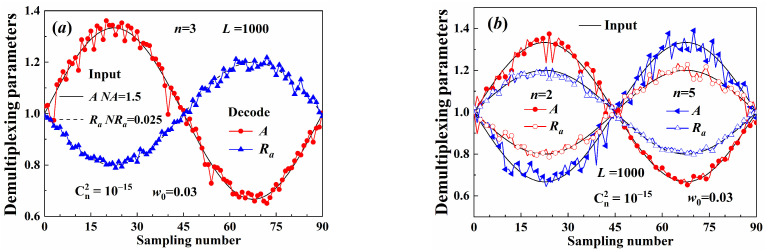
Demultiplexing parameters: (**a**) single beam, (**b**) multiplex beams.

Figure 13 illustrates the demultiplexing results for different Gv beams. The multiplexing input information consisted of cosine signals carried by both the beam aperture and the beam amplitude. The period of the input signal was 90. The multi-parameters were decoded at different sampling times, as shown in Figure 13. The decoding parameter was normalized and equaled the initial value divided by the mean of the sine signal in one period. The normalized constants of the input are noted in Figure 13a and represented as the average amplitude NA and the average aperture radius $NR_{a}$, separately. The decoding amplitude and the aperture radius were both consistent with the input values, as shown in Figure 13. The decoding precision of the aperture radius was also higher than that of the amplitude. Moreover, the demultiplexing method can also decode the signal carried by multiplex beams with different coaxially OAM values, as shown in Figure 13b. Therefore, the aperture radius can be applied as the information carrier, and signals carried were less influenced by the turbulence.

## 4. Conclusions

In this study, a multiplexing method for truncated Gv beams was proposed. The aperture radius as a new information carriers was provided, and it could be controlled by an external variable aperture.

The demultiplexing method of aperture-modulated OAM beams was also improved. The field of the beams propagating through the turbulence was derived and discretized with Gauss–Legendre quadrature formulas, and it was equal to the field simulated using the phase screen method. By solving the field equations at different radii using the least squares fitting method, the beam OAM states, amplitude, Gaussian spot radius, and aperture radius were demultiplexed. The decoding result coincided well with the input values. Moreover, an iterative method to search the center of the distortion beam was also provided. And this method can could be applied to correct the decoding error caused by the beam wander. When the aperture size was less than the beam spot size, the aperture radius can be decoded. The decoding precision of the beam width was greater than that of the complex amplitude, because the turbulence introduced perturbations in the complex amplitude. Given the same input, the decoding precision of the aperture radius of beams with a constant Gaussian spot radius was higher than that of the Gaussian radius of the beams with constant aperture radius. The decoding precision was also more sensitive to the truncated aperture radius. Therefore, the aperture radius is recommended as the extra dimension for the multiplexing communication. Moreover, the sinusoidal signal carried by the multi-coaxial beams with different OAM was also decoded, showing good agreement with the input information.

This study on OAM beam multiplexing provides a simple way to modulate the information carried by the OAM beams, and it has promising application on the large capacity laser communication.

## Data Availability

The data that support the findings of this study are available from the corresponding author upon reasonable request.
